# Effect of Omega-3 Fatty Acid Alone and in Combination with Proprietary Chromium Complex on Endothelial Function in Subjects with Metabolic Syndrome: A Randomized, Double-Blind, Parallel-Group Clinical Study

**DOI:** 10.1155/2021/2972610

**Published:** 2021-06-25

**Authors:** Usharani Pingali, Chandrasekhar Nutalapati, Srinivas Gundagani

**Affiliations:** Department of Clinical Pharmacology & Therapeutics, Nizam's Institute of Medical Sciences, Panjagutta, Hyderabad 500082, Telangana, India

## Abstract

Metabolic syndrome (MetS) represents a cluster of metabolic abnormalities that include hypertension, central obesity, insulin resistance, and dyslipidemia and is strongly associated with an increased risk of diabetes, cardiovascular diseases (CVD), and all-cause mortality. Early diagnosis is important to employ lifestyle and risk factor modification. Existing therapies are limited. Studies report positive effect of omega-3 fatty acids (*ω*-3FA) on symptoms of metabolic syndrome. The present study was undertaken to evaluate the effect of *ω*-3FA alone and in combination with proprietary chromium complex (PCC) on endothelial function in subjects with metabolic syndrome. In this randomized, double-blind, parallel-group study, subjects were enrolled into the study after ethics committee (EC) approval and informed consent. Eligible subjects were randomized to receive *ω*-3FA concentrate 2000 mg (Group A-18 subjects), *ω*-3FA concentrate 2000 mg + PCC200 mcg (Group B-19 subjects), and *ω*-3FA concentrate 2000 mg + PCC400 mcg (Group C-21 subjects) daily for 12 weeks. Endothelial dysfunction as measured by reflection index (RI), biomarkers of oxidative stress (NO, MDA, and glutathione), and inflammation (hsCRP, endothelin-1, ICAM-1, and VCAM-1) were evaluated at baseline, 4, and 12 weeks. Lipid-profile and platelet-aggregation tests were performed at baseline and 12 weeks. Adverse drug reactions were recorded. Compliance was assessed by pill count method. GraphPad Prism8 was used for statistical analysis. Significant changes were seen from 4 weeks onwards in all the parameters evaluated. Significant improvement in RI% (mean ± SD = −2.56 ± 0.77 to −3.27 ± 0.67-group A, −2.33 ± 0.76 to 4.72 ± 0.79-group B; −2.39 ± 1.13 to 6.46 ± 1.00-group C) was seen at 12 weeks. Significant improvement in biomarkers of oxidative stress and inflammation was seen with all the treatment groups. Similarly, significant improvement in lipid profile was seen in group B and group C, while group A showed change in HDL, VLDL, and TG. Group C demonstrated the best response in the parameters evaluated. Three patients in group C reported gastrointestinal adverse events, which resolved spontaneously; none stopped the therapy. So, the addition of PCC to *ω*-3FA may prove to have beneficial effect in reducing cardiovascular morbidity in MetS patients.

## 1. Introduction

Metabolic syndrome (MetS) is defined by a constellation of interconnected physiological, biochemical, clinical, and metabolic factors that directly increases the risk of cardiovascular disease, type 2 diabetes mellitus, and all-cause mortality [1, 2]. Insulin resistance, visceral adiposity, atherogenic dyslipidemia, endothelial dysfunction, genetic susceptibility, elevated blood pressure, hypercoagulable state, and chronic stress are the several factors that constitute the syndrome [3]. It is a significant and escalating public health and clinical challenge worldwide in the wake of urbanization, surplus energy intake, increasing obesity, and sedentary life habits. MetS increases the risk of T2DM, CVD [4], stroke and MI, and dying from such event [5] regardless of a previous history of cardiovascular events [6].

A strong association between MetS diagnosed according to the Adult Treatment Panel (ATP) III criteria [7] and oxidative stress has been found. Hyperglycemia and inflammation, important components of MetS, increase the production of reactive oxygen species (ROS) [8], resulting in increased oxidative stress with overactivation of NADPH oxidase. This process reduces the bioavailability of nitric oxide (NO) [9], explaining both the low NO levels in subjects with metabolic disorders and the correlation of NO levels with body mass index (BMI), blood pressure, and triglyceridemia [10]. Thus, decreased NO availability appears to play a significant role in MetS. Glutathione (GSH) is one of the essential antioxidant mechanisms in living organisms. Low GSH levels are associated with oxidative stress and the manifestation of various diseases [11]. Similarly, malondialdehyde (MDA) is a biomarker of intensified lipid peroxidation, which leads to the production of free radicals. Studies have shown that increased oxidative stress through lipid peroxidation may lead to diabetes [12].

Studies have shown that population with MetS have increased levels of inflammatory markers like hsCRP. There also seems to be an association of hsCRP with adhesion molecules such as intercellular adhesion molecule-1 (ICAM-1) and vascular cell adhesion molecule-1 (VCAM-1) [13]. Circulating levels of both soluble ICAM-1 and VCAM-1 have been reported in MetS. The levels of soluble adhesion molecules like sICAM-1 and sVCAM-1 reflect the expression of membrane-bound adhesion molecules and the process of vascular inflammation of the vessel wall [14]. Similarly, endothelin-1 (ET-1), which is linked to vascular inflammation, may also play an essential role in the development of arteriosclerosis along with adhesion molecules. It also seems to have vasoconstrictor activity. So, increased production or activity of endothelin-1 may lead to MetS [15]. Data on such biomarkers in subjects with MetS is scanty.

Studies report that excess cholesterol substantially alters the endothelial function, leading to a decreased relaxation of the arterial vessels [16, 17]. The defect has been described mainly in atherosclerotic subjects, as well as in asymptomatic subjects with hypercholesterolemia [18]. Data from the MRFIT study [19] show that the relationship between cholesterol and coronary artery disease is active and graded across all levels of blood pressure; similarly, the relationship between blood pressure and coronary artery disease is substantial and graded across all levels of cholesterol.

Despite advancement in our understanding of metabolic syndrome currently, there is no single therapy approved to manage it as a single condition, as it is a multifaceted health problem. The mainstay of treatment includes a healthy lifestyle, which may include promoting physical activity for weight reduction and a balanced diet. These are likely to be the most effective in controlling metabolic syndrome; nonetheless, it is challenging to initiate and maintain a healthy lifestyle. Pharmacotherapy may include agents that deal with different components of MetS like obesity, diabetes, hypertension, and dyslipidemia that are used singly or in combination: antiobesity drugs, thiazolidinediones, metformin, statins, fibrates, renin-angiotensin system blockers, glucagon-like peptide-1 agonists, sodium-glucose transporter-2 inhibitors, and some antiplatelet agents such as cilostazol. Nevertheless, multidrug treatment may lead to drug interactions, decreases patient compliance, and increases health costs, so it becomes relevant to introduce single therapies for treating different aspects of MetS. In such a scenario, nutraceutical may have an important role to play [20–23].

Omega-3 polyunsaturated fatty acids (PUFAs), such as eicosapentaenoic acid and docosahexaenoic acid, are widely regarded as cardioprotective. Large-scale, randomized clinical trials have reported that dietary intake of omega-3 PUFAs improves the prognosis of patients with symptomatic heart failure or recent myocardial infarction [24]. Animal studies have shown that omega-3 fatty acid may possess potent antioxidant and anti-inflammatory properties and may prevent fibrosis of different organs [25]. It also empowers the antioxidant defense system by reducing oxidative stress and increasing the activities of the major antioxidant enzymes such as superoxide dismutase, catalase, and glutathione peroxidase [26]. The American Heart Association recommends that all adults eat fish 2 to 3 times per week because of its content of *ω*-3FA. *ω*-3FA has been shown to have positive cardiovascular effects including lowering triglycerides, blood pressure, and platelet aggregation, increasing arterial compliance and improving endothelial function [27].

Previous studies done at our department had shown that a proprietary chromium complex (PCC) improved endothelial function, biomarkers of oxidative stress, and lipid profile significantly [28]. PCC is prepared by complexation of Cr^3+^ chloride with an aqueous extract of *Phyllanthus emblica* fruits, which contain polyphenolic compounds, and purified Shilajit, a herbomineral product, which, by virtue of its liposomal fulvic acid content, increases the bioavailability of chromium. PCC has been shown to have synergistic activity to improve endothelial function and cardiovascular health (US Patent 10,183,047). Studies report that Shilajit induces the growth of blood vessels [29] and has a prominent cardioprotective effect [30]. In a study done by us [31], we had evaluated the effect of fish oil alone and in combination with different doses of proprietary chromium complex (PCC) in type 2 diabetes patients. We had demonstrated that fish oil, in combination with PCC, significantly improved endothelial function. The present prospective, randomized, double blind, parallel group study was, thus, undertaken to evaluate the effect of combining *ω*-3FA alone and with two doses of PCC (10 mg & 20 mg) versus *ω*-3FA alone on endothelial function, systemic inflammation, vascular inflammation, and lipid profile in subjects with metabolic syndrome. Furthermore, the study was designed to evaluate study medications in improving the various parameters of metabolic syndrome.

## 2. Materials and Methods

This 12-week prospective, randomized, double-blind, parallel-group study was conducted in the Department of Clinical Pharmacology and Therapeutics at NIMS, Hyderabad, India, between the period from November 2017 to June 2018.

### 2.1. Enrollment of Study Subjects

The subjects were recruited from the Outpatient Department of General Medicine. The participants were provided with the subject information sheet, informed consent form, and contact information for study enrollment.

#### 2.1.1. Inclusion Criteria

These include subjects of either sex aged 30–65 years having endothelial dysfunction defined as ≤6% change in reflection index (RI) on postsalbutamol challenge test and those complying with “The International Diabetes Federation” guidelines, dated 2017 [32], which states central obesity (defined as waist circumference with ethnicity-specific values – if BMI is >30 kg/m^2^, central obesity can be assumed, and waist circumference does not need to be measured), and any two of the following: raised triglycerides >150 mg/dL (1.7 mmol/L) or specific treatment for this lipid abnormality, reduced HDL cholesterol: <40 mg/dL (1.03 mmol/L) in males, <50 mg/dL (1.29 mmol/L) in females, or specific treatment for this lipid abnormality, raised blood pressure: systolic BP>130 or diastolic BP >85 mmHg, or treatment of previously diagnosed hypertension, raised fasting plasma glucose of ≥100 mg/dL, previously diagnosed type 2 diabetes were included into the study.

#### 2.1.2. Exclusion Criteria

These include subjects with abnormal hematological, or biochemical parameters considered significant by investigator, uncontrolled hypertension, hypertriglyceridemia, impaired hepatic or renal functions, history of malignancy or stroke, history of smoking/alcoholism, any other severe disease requiring active treatment, and on any other dietary or herbal supplements.

### 2.2. Ethical Consideration

**Figure 1:**
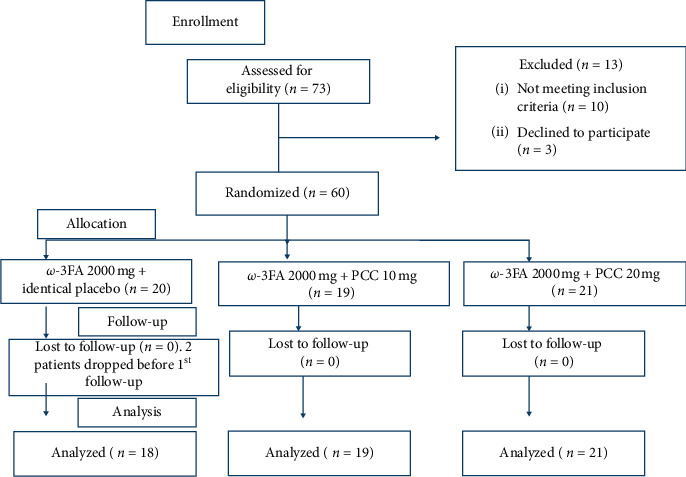
Consort flow diagram of the study.

The present study was approved by Nizam's Institute of Medical Sciences-Institutional Ethics Committee. Written informed consent was taken from all the subjects before participation in the study. This trial was conducted in accordance with the Declaration of Helsinki [33].  Figure 1 depicts Consolidated Standards of reporting Trials (CONSORT) flow chart. This study was registered with Clinical Trials Registry, India (CTRI) with the registration number of https://CTRI/2018/05/013548; date: 01/05/2018. Retrospectively registered. http://ctri.nic.in/Clinicaltrials/rmaindet.php?trialid=23364&EncHid=82660.16614&modid=1&compid=19.

### 2.3. Investigational Products

#### 2.3.1. Product 1

Each soft gelatin capsule of Product 1 contains 1000 mg of a refined *ω*-3FA, having 300 mg of EPA (TG) and 200 mg of DHA (TG), and 10 mg of microcrystalline cellulose (excipient).

#### 2.3.2. Product 2

Each soft gelatin capsule of Product 2 contains 1000 mg of a refined *ω*-3FA, containing 300 mg of EPA (TG) and 200 mg of DHA (TG), and 10 mg of PCC, also known as “Crominex®,” (equivalent to 200 mcg of Cr^3+^).

Both products were made at Tishcon Corp., NY, and supplied by Natreon, NJ.

### 2.4. Randomization and Blinding

A total of 73 subjects were screened out, of which 60 subjects meeting the inclusion criteria were enrolled in the study. They were randomized into one of the three treatment groups in a double-blind fashion using computer-generated block randomization. Two subjects dropped out of the study before the first follow-up, citing logistical reasons. A total of 58 subjects completed the study.

All the capsules given to the subjects were of similar size, color, shape, and texture. Each subject was given a sealed packet, which was sequentially designated numbers and containing two sealed bottles, each bottle containing 33 capsules. The packets were dispensed by the pharmacist to the subjects as per the randomly allocated sequence. They were instructed to take one capsule from each bottle daily morning with water after breakfast for a period of 4 weeks. Thereafter, at each visit, the subject was given a new sealed packet containing two sealed bottles and asked to consume in the same pattern as instructed earlier.

The pharmacist provided the subjects with bottles containing the study products, which were sequentially numbered. The bottles were dispensed as per the randomly allocated sequence. The principal investigator and the subjects were blinded. The allocations were unblinded after the completion of the study period for the purpose of the tabulation of data and statistical analysis.

### 2.5. Interventions

All the randomized subjects received the following interventions as per the groups allotted to them.

#### 2.5.1. Group A

They take two soft gelatin capsules of Product 1 once a day, to provide a total dose of 2000 mg of *ω*-3FA, containing 600 mg of EPA + 400 mg of DHA.

#### 2.5.2. Group B

They take one soft gelatin capsule of Product 1 + One soft gelatin capsule of Product 2 once a day to provide a total dose of 2000 mg of *ω*-3FA, containing 600 mg of EPA + 400 mg of DHA and 10 mg of PCC (equivalent to 200 mcg of Cr^3+^).

#### 2.5.3. Group C

They take two soft gelatin capsules of Product 2 once a day to provide a total of 2000 mg of *ω*-3FA, containing 600 mg of EPA + 400 mg of DHA and 20 mg of PCC (equivalent to 400 mcg of Cr^3+^).

### 2.6. The Efficacy Variables

The primary efficacy measures were improvement in endothelial function as assessed by more than 6% change in reflection index at 12 weeks in all the treatment groups, improvement in endothelial function and oxidative stress biomarkers (NO, GSH, and MDA), inflammation biomarker (hsCRP), vascular inflammation biomarkers (ET-1, ICAM-1, and VCAM-1), glycosylated haemoglobin (HbA1c), platelet aggregation, and lipid profile. Secondary efficacy measure was the safety and tolerability assessment of the study products.

### 2.7. Assessment of Endothelial Function

Endothelial function was assessed using the salbutamol challenge test employing digital volume plethysmography, following the methods of Chowienczyk et al. [34] and Naidu et al., [35]. The patients were inspected at the supine position after resting for 5 minutes. A digital volume pulse (DVP) was recorded using a photo-plethysmograph (Pulse Trace PCA2, PT200, Micro Medical, Kent, UK) transmitting infrared light at 940 nm, placed on the index finger of the right hand. The signal from the plethysmograph was digitized using a 12-bit analog to a digital converter with a sampling frequency of 100 Hz. DVP waveforms were recorded over 20-second period, and the height of the late systolic/early diastolic portion of the DVP was expressed as a percentage of the amplitude of the DVP to yield the reflection index (RI), following the procedure of Millasseau et al. [36]. After the DVP recording, three measurements of reflection index (RI) were calculated, and the mean value was determined. The patients were then administered with 400 *μ*g of salbutamol by inhalation. After 15 minutes, three more measurements of RI were recorded, and the difference in mean RI before and after administration of salbutamol was used for assessing endothelial function. A change in ≤6% of RI postsalbutamol administration was considered as endothelial dysfunction.

### 2.8. Biomarker Evaluation

Nitric oxide [37, 38] (by colorimetric detection with Griess reagents), MDA [39] (by thiobarbituric acid reactive substance test), and GSH (by Ellman's method) [40] levels were estimated spectrophotometrically. The ELISA method was employed to assess hsCRP, ET-1, ICAM-1, and VCAM-1 levels. Blood samples were collected at the department from the subjects after overnight fasting for the estimation of haemoglobin, blood urea and serum creatinine, liver function tests, and lipid profile, which included total cholesterol (TC), high-density lipoprotein cholesterol (HDL-C), low-density lipoprotein cholesterol (LDL-C), very low-density lipoprotein cholesterol (VLDL-C), and TGs, using appropriate standard techniques. A platelet aggregation test with 10 *μ*M/mL ADP and 2 *μ*g/mL collagen was performed by employing a platelet aggregometry test (Chrono-log light transmittance aggregometry).

Serum sample was used to estimate biomarkers (MDA, NO, GSH, hsCRP, ICAM-1, VCAM-1, and ET-1) and other biochemical tests. Whole blood samples were collected in EDTA tubes and were used for estimating HbA1c. The analysis of biomarkers (MDA, NO, GSH, hsCRP, ICAM-1, VCAM-1, and ET-1) was performed at the end of the study period in one batch. After collection of the blood samples, serum was separated and stored at −80 degrees centigrade. All the tests were performed at our institutional laboratory.

### 2.9. Follow-Up Visits

Salbutamol challenge test employing digital volume plethysmography was used to assess endothelial function as estimated by RI at every visit (baseline, 4, 8, and 12 weeks). Blood samples were collected, after an overnight fast, for evaluation of MDA, NO, GSH, hsCRP, ICAM-1, VCAM-1, and ET-1, at baseline, 4, and 12 weeks. Lipid profile, HbA1c, and platelet aggregation were evaluated at baseline and at the end of 12 weeks.

### 2.10. Safety Evaluation

A complete physical examination was done at each visit. Safety laboratory investigations for hematological, hepatic, and renal biochemical parameters were evaluated at baseline and the end of the study, and if necessary, during the study. Subjects were enquired for the presence of adverse drug reactions (ADR) at every visit. ADRs were recorded in the case report form if any.

### 2.11. Compliance Verification

Pill-count method was used for the assessment of compliance with the study medications. Compliance was considered good if a patient received >80%, fair between 60% and 80%, or poor if <60% of the dispensed medication.

### 2.12. Sample Size Determination

A total sample size of 75 was estimated to be needed to enroll 66 subjects, assuming a pooled standard deviation of 2 units to achieve a power of 80%, 5% significance, screen failure 10%, and a dropout rate of 10%.

### 2.13. Statistical Analyses

Study data are expressed as mean ± SD. “Paired *t*-test” was used to compare the change in biomarkers from baseline to post-treatment within groups, and ANOVA for between group comparisons. Post hoc analysis between the groups was done using Tukey's test. A *p*-value of less than 0.05 was considered statistically significant. All statistical analysis was performed using licensed version of the software GraphPad Prism8.

## 3. Results

From a total of 73 screened subjects, 60 eligible subjects were randomized in the study by computer generated block randomization and 58 completed 12 weeks of treatment. Two subjects dropped out of the study before the first follow-up. A total of 18 subjects in Group A, 19 in Group B, and 21 in Group C completed 12 weeks of treatment.  Table 1 depicts the demographic characteristics of the three groups. There were no significant differences between treatment groups in baseline characteristics, including age and body mass index (BMI), indicating a homogenous population.

### 3.1. Endothelial Function (RI%)

Endothelial function was assessed by evaluating RI. As seen from Table 2, all the groups have shown significant improvement in RI, indicating an improvement in endothelial function at 12 (*p* ≤ 0.0001), 8 (*p* ≤ 0.0001) as well as 4 (*p* ≤ 0.05) weeks. Further analysis showed that treatment with Group C was significantly better than Group B and Group A at 12 weeks (*p* ≤ 0.001).

### 3.2. Endothelial Marker

A statistically significant difference in mean % change was seen in the levels of NO (+5.15, +10.11%, +25.14%) as depicted in Table 3.

### 3.3. Oxidative Stress Markers

The biomarkers of oxidative stress showed improvement at 12 weeks with all the groups. As depicted in Table 3, statistically significant difference in mean % change was seen: MDA (−0.74%, −6.74%, −18.46%), and GSH (+1.01, +4.96%, +15.39%).

### 3.4. Inflammation Marker

The results were similar, as shown in Table 3 with the markers of inflammation at 12 weeks: hsCRP (−1.39%, −5.03%, −21.56%), ICAM-1 (−8.84%, −16.24%, −23.57%), VCAM-1 (−3.58%, −17.58%, −31.62%), and ET-1 (−8.45%, −12.37%, −26.89%). Group B was better than Group A (*p* < 0.01), while Group C was more efficacious than the other two groups (*p* < 0.001). There was no significant difference between Groups A and B in improving the ET-1 levels.

### 3.5. Lipid Profiles

As shown in Table 4, at 12 weeks, statistical significance was seen with all the three groups in mean % change of HDL (1.43%, 4.98%, 9.55%), VLDL (−2.28%, −7.30%, −13.15%), and TG (−2.33%, −6.87%, −13.92%), while Groups B and C have also shown significant mean % change in the levels of TC (−3.48%, −6.94%) and LDL-C (−5.12%, −10.98%). The significance with Group A was *p* < 0.05, while Group B and Group C had shown a significance of *p* < 0.0001 when compared to baseline. Between-group analysis showed Group C to be better than Group A (*p* < 0.001), as well as Group B (*p* < 0.01).

### 3.6. Effect on Glycosylated Haemoglobin A1c (HbA1c %)

A statistically significant improvement (*p* ≤ 0.01) in HbA1c was seen with only Group C from 6.17 to 5.89, which was a modest improvement.

### 3.7. Platelet Aggregation

No effect was recorded on platelet aggregation with any of the groups.

### 3.8. Safety Evaluation

All safety haematological and biochemical parameters were within normal limits with all the groups at the end of the treatment. Three subjects in Group C complained of gastrointestinal intolerance, and one patient reported nausea, while two reported dyspepsia, which subsided with symptomatic treatment. However, no subject in any of the groups discontinued the study due to adverse events.

## 4. Discussion

Significant, though modest, improvement in endothelial function, biomarkers of oxidative stress, systemic and vascular inflammation, and lipid profile was seen with *ω*-3FA, but the addition of PCC, especially at 20 mg dose (400 mcg of Cr^3+^), significantly increased the efficacy of *ω*-3FA.

In the present study, RI was measured to assess endothelial function, using salbutamol challenge test employing digital volume plethysmography (DPG). This test is a simple, noninvasive, reproducible and a reliable method [35]. It does not require trained personnel, unlike flow-mediated vasodilation (FMD), which requires sophisticated instrument and a skilled operator and has a chance for large interindividual variability in the assessment of ED [41]. In a study done by Skulas Ray et al. [42] on subjects with moderate hypertriglyceridemia, two different doses of *ω*-3FA (0.85 and 3.4 g/day) taken for eight weeks showed no effect on endothelial function. In their study, baseline endothelial function testing was absent. In another study by Wong et al. [43], 12-week therapy with *ω*-3FA (4 g/day) in type 2 DM, no improvement in endothelial function was noted. In a previous study [28] done at our department, we have shown the beneficial effect of PCC on endothelial function in T2DM patients. In the present study, we have shown that the addition of PCC, especially at 20 mg (400 mcg of Cr^3+^), significantly enhanced the efficacy of *ω*-3FA in improving endothelial function in MetS subjects, unlike the study reported by Wong et al. We assume that highly significant improvement in endothelial function can be attributed to the combination of *ω*-3FA with PCC.

Malondialdehyde (MDA) is a byproduct formed by lipid peroxidation due to free radical generation. We have shown a significant reduction in MDA with all the groups. The combination of *ω*-3FA + 20 mg of PCC had shown better results when compared to the other two groups. Our results are similar to those of Shifdar et al. [44], who have related the potential mechanism for the decrease in MDA to the assembly of omega-3 fatty acids in membrane lipids and lipoproteins making the double bonds less available for free radical attack, inhibition of the prooxidant enzyme phospholipase A2 and stimulation of antioxidant enzymes. So, we assume that combination of *ω*-3FA with PCC seems to have a more potent effect than *ω*-3FA alone.

In a 12-week study by Asemi et al. [45], *ω*-3FA alone and its combination with alpha-tocopherol significantly increased NO levels with no change in GSH levels. They have attributed increased levels of NO to direct and indirect effects of *ω*-3FA on the arterial wall. In our study, we reported highly significant improvement in NO and GSH levels at the end of 12 weeks with all the treatment groups, with Group C showing better results than the other two groups. The probable mechanism in improved NO levels could be as mentioned above. The improvement in GSH levels could be attributed to the fact that *ω*-3FA improves levels of glutathione reductase (GR) [46]. As previously stated, the highly significant improvement in Group B and Group C can be due to the combination of *ω*-3FA with PCC, which seems to be more beneficial than *ω*-3FA alone.

Yamada et al. [47] observed that EPA reduced the levels of both ICAM-1 and VCAM-1 after three months of treatment in metabolic syndrome subjects. These results are in agreement with the highly significant reduction in ICAM-1, VCAM-1, and ET-1 levels in all the 3 treatment groups in the present study, with the best improvement in Group C. This effect may be attributed to the inhibitory effect of *ω*-3FA on the endothelial expression of adhesion molecules [48]. The addition of PCC to *ω*-3FA seems to have provided a highly significant response on biomarkers of vascular inflammation, when compared to *ω*-3FA given alone.

In previous studies [49, 50] done at our department, we have shown the beneficial effects of PCC and its constituent materials on hsCRP. In a study by Schiano et al. [51], *ω*-3FA did not show any effect on hsCRP levels. Similarly, Mackay et al. [52] reported no change in hsCRP and platelet aggregation with *ω*-3FA. However, in the present study, we report significant changes in hsCRP levels, at 12-weeks, even with *ω*-3FA alone (Group A), while Groups B and C had shown highly significant improvement. Such a result may be due to addition of PCC to *ω*-3FA. We report no change in platelet aggregation, similar to the study by Mackay et al.

Koh et al. [53], in their study on subjects with hypertriglyceridemia, reported that *ω*-3FA reduced TG levels. Similarly, JELIS study by Yokoyama [54] had reported improvement in TC and HDL levels. Two other studies by Cicerio and Colletti [55] in hypertriglyceridemia subjects and Sawada et al. [56] in subjects with impaired glucose metabolism and coronary artery disease reported improvement in TG, HDL-C, and LDL-C levels. They related this improvement in lipid parameters to improvement in endothelial dysfunction. Similar to these studies, we report significant improvement in all the parameters of lipid profile with *ω*-3FA + PCC (Groups B and C). Group A had improved the levels of HDL-C, VLDL-C, and TG extremely slightly.

## 5. Conclusion

Omega-3 fatty acid (*ω*-3FA) alone and in combination with PCC 10 mg (200 mcg Cr^3+^) and 20 mg (400 mcg Cr^3+^) had a marked effect on endothelial function, accompanied by a significant improvement in the biomarkers NO, GSH, MDA, hsCRP, ICAM-1, VCAM-1, and ET-1, but the improvement was only modest with *ω*-3FA alone, but highly significant with *ω*-3FA and PCC combinations. There was a marked improvement in the lipid profile as well, and again the best improvement was seen with *ω*-3FA + PCC 20 mg (400 mcg Cr^3+^), which is also the only group to show modest improvement in HbA1c. All the study medications were well tolerated, and no serious adverse events were observed. None of the subjects discontinued the study due to any adverse event suggesting the favorable safety profile of the treatments.

This study suggests that combining *ω*-3FA with PCC may improve the cardiovascular morbidity in metabolic syndrome subjects in a dose-dependent manner. Further studies are warranted to substantiate the beneficial effect of this combination therapy when used as adjunctive therapy in subjects with metabolic syndrome. The limitation of this study is that it did not have a placebo group.

## Figures and Tables

**Table 1:** Baseline characteristics of subjects.

Parameter	*ω*-3FA 2000 mg (A) (*n* = 18)	*ω*-3FA 2000 mg + PCC 10 mg (B) (*n* = 19)	*ω*-3FA 2000 mg + PCC 20 mg (C) (*n* = 21)
Age in yrs	50.89 ± 4.34	52.58 ± 4.57	54.33 ± 6.41
Sex (M/F)	11/7	12/7	13/8
Bodyweight (kg)	79.78 ± 3.57	80.92 ± 3.70	80.08 ± 4.41
Height (cm)	161.0 ± 4.84	161.4 ± 4.89	160.9 ± 5.20
BMI (kg/m^2^)	30.78 ± 0.79	31.02 ± 0.73	30.94 ± 0.71

Values expressed as mean ± SD. SD: standard deviation. BMI: Body Mass Index.

**Table 2:** Effect of treatment on reflection index % (measure of endothelial function).

Parameter	*ω*-3FA 2000 mg (A) (*n* = 18)				*ω*-3FA 2000 mg + PCC 10 mg (B) (*n* = 19)				*ω*-3FA 2000 mg + PCC 20 mg (C) (*n* = 21)			
RI (%)	Baseline	4 weeks	8 weeks	12 weeks	Baseline	4 weeks	8 weeks	12 weeks	Baseline	4 weeks	8 weeks	12 weeks
Mean ± SD	−2.56 ± 0.77	−2.67 ± 0.70	−2.72 ± 0.73	−3.27 ± 0.67	−2.33 ± 0.76	−3.16 ± 0.75	−3.87 ± 0.75	−4.72 ± 0.79	−2.39 ± 1.13	−3.45 ± 1.14	−4.48 ± 1.02	−6.46 ± 1.00
*p* value	—	≤0.05	≤0.05	≤0.0001	—	≤0.0001	≤0.0001	≤0.0001	—	≤0.001	≤0.0001	≤0.0001

Values expressed as mean ± SD. Ns: not significant. Within groups from baseline to each week (paired *t*-test was done): 4 wks–A *p* ≤ 0.05, B *p* ≤ 0.0001, C *p* ≤ 0.001.8 wks-A *p* ≤ 0.05, B & C *p* ≤ 0.0001.12 wks–A, B & C *p* ≤ 0.0001. Between groups (ANOVA and Tukey's post hoc test was done): 4 wks–B vs A *p* ≤ 0.05, C vs A *p* ≤ 0.01, C vs B ns. 8 wks-B vs A & C vs A *p* ≤ 0.001, C vs B ns. 12 wks–B vs A, C vs A & C vs B *p* ≤ 0.001.

**Table 3:** Effect of treatment on biomarkers of oxidative stress, systemic inflammation, and vascular inflammation at 12 weeks.

Parameter (normal range)	*ω*-3FA 2000 mg (A) (*n* = 18)				*ω*-3FA 2000 mg + PCC 10 mg (B) (*n* = 19)				*ω*-3FA 2000 mg + PCC 20 mg (C) (*n* = 21)			
	Baseline	12 weeks	Mean change (%)	*p* value baseline to 12 weeks	Baseline	12 weeks	Mean change (%)	*p* value baseline to 12 weeks	Baseline	12 weeks	Mean change (%)	*p* value baseline to 12 weeks
MDA (2.70–4.50 nM/ml)	3.70 ± 0.31	3.66 ± 0.24	−0.74	*p* ≤ 0.0001	3.65 ± 0.31	3.40 ± 0.26	−6.74	*p* ≤ 0.0001	4.46 ± 0.40	3.64 ± 0.38	−18.46	*p* ≤ 0.0001
NO (24.8748.23 *μ*M/L)	31.54 ± 3.80	33.10 ± 3.54	+5.15	*p* ≤ 0.0001	30.66 ± 3.6516	33.65 ± 3.10	+10.11	*p* ≤ 0.0001	29.74 ± 2.59	37.14 ± 2.80	+25.14	*p* ≤ 0.0001
GSH (509–657 *μ*M/L)	548.01 ± 15.64	553.53 ± 15.79	+1.01	*p* ≤ 0.0001	553.84 ± 12.46	581.27 ± 13.71	+4.96	*p* ≤ 0.0001	558.65 ± 17.84	644.58 ± 25.08	+15.39	*p* ≤ 0.0001
hsCRP (<1.0 mg/L–low risk 1.0–3.0 mg/L average risk >3.0 mg/L–high risk)	3.09 ± 0.12	3.05 ± 0.10	−1.39	*p* ≤ 0.05	3.33 ± 0.19	3.16 ± 0.22	−5.03	*p* ≤ 0.0001	3.19 ± 0.21	2.50 ± 0.23	−21.56	*p* ≤ 0.0001
ICAM-1 (302 1115 ng/ml)	560.17 ± 132.90	512.78 ± 129.81	−8.84	*p* ≤ 0.0001	591.21 ± 155.36	497.84 ± 140.83	−16.24	*p* ≤ 0.0001	633.62 ± 153.85	488.95 ± 145.70	−23.57	*p* ≤ 0.0001
VCAM-1 (400.6–1340.8 ng/ml)	702.11 ± 123.66	678.39 ± 127.01	−3.58	*p* ≤ 0.0001	730.14 ± 151.72	597.93 ± 110.74	−17.58	*p* ≤ 0.0001	756.67 ± 142.94	514.17 ± 89.96	−31.62	*p* ≤ 0.0001
ET-1 (3.55 ± 1.78 pg/mL)	3.98 ± 0.25	3.64 ± 0.23	−8.45	*p* ≤ 0.0001	4.04 ± 0.50	3.54 ± 0.48	−12.37	*p* ≤ 0.0001	4.23 ± 0.45	3.07 ± 0.27	−26.89	*p* ≤ 0.0001

Values expressed as mean ± SD. Ns: not significant. Within groups from baseline to each week (paired *t*-test was done): MDA: A *p* ≤ 0.001, B & C *p* ≤ 0.0001; NO: A, B & C *p* ≤ 0.0001; GSH: A, B & C *p* ≤ 0.0001; hsCRP: A *p* ≤ 0.05, B & C *p* ≤ 0.0001; ICAM-1: A, B & C *p* ≤ 0.0001; VCAM-1: A, B & C *p* ≤ 0.0001; ET-1: A, B & C *p* ≤ 0.0001. Between groups (ANOVA and Tukey's post hoc test was done): MDA: B vs A *p* ≤ 0.01, C vs A & C vs B *p* ≤ 0.001; NO: B vs A *p* ≤ 0.01, C vs A & C vs B *p* ≤ 0.001; GSH: B vs A, C vs A & C vs B *p* ≤ 0.001; hsCRP: B vs A *p* ≤ 0.01, C vs A & C vs B *p* ≤ 0.0001; ICAM-1: B vs A *p* ≤ 0.01, C vs A & C vs B *p* ≤ 0.001; VCAM-1: B vs A, C vs A & C vs B *p* ≤ 0.001; ET-1: B vs A ns, C vs A, C vs B *p* ≤ 0.001.

**Table 4:** Effect of treatment on lipid profile at 12 weeks.

Parameter (normal range) (mg/dl)	*ω*-3FA 2000 mg (A) (*n* = 18)				*ω*-3FA 2000 mg + PCC 10 mg (B) (*n* = 19)				*ω*-3FA 2000 mg + PCC 20 mg (C) (*n* = 21)			
	Baseline	12 weeks	Mean change (%)	*p* value baseline to 12 weeks	Baseline	12 weeks	Mean change (%)	*p* value baseline to 12 weeks	Baseline	12 weeks	Mean change (%)	*p* value baseline to 12 weeks
Total cholesterol (130200)	185.83 ± 16.11	185.44 ± 15.26	−0.12	ns	182.32 ± 13.91	176.05 ± 14.90	−3.48	*p* ≤ 0.0001	197.33 ± 25.62	188.33 ± 22.93	−6.94	*p* ≤ 0.0001
HDL-C (>40)	41.00 ± 5.47	42.17 ± 6.53	1.43%	*p* ≤ 0.05	39.26 ± 4.48	41.21 ± 4.67	4.98	*p* ≤ 0.0001	42.57 ± 5.77	46.52 ± 5.62	9.55	*p* ≤ 0.0001
LDL-C (≤130)	102.22 ± 16.38	101.61 ± 14.59	−0.11	ns	95.63 ± 8.80	90.79 ± 9.48	−5.12	*p* ≤ 0.0001	115.67 ± 23.71	102.71 ± 21.51	−10.98	*p* ≤ 0.0001
VLDL-C (≤30)	42.61 ± 9.17	41.67 ± 9.35	−2.28	*p* ≤ 0.05	47.42 ± 7.71	44.05 ± 7.81	−7.30	*p* ≤ 0.0001	39.10 ± 6.12	34.10 ± 6.40	−13.15	*p* ≤ 0.0001
Triglycerides (≤150)	213.00 ± 46.11	208.17 ± 46.82	−2.33	*p* ≤ 0.01	236.95 ± 3 9.05	220.95 ± 38.36	−6.87	*p* ≤ 0.0001	195.81 ± 30.78	169.38 ± 32.33	−13.92	*p* ≤ 0.0001

Values expressed as mean ± SD. Within groups from baseline to each week (paired *t*-test was done): TC: A–ns, B & C-*p* ≤ 0.0001; HDL-C: A-*p* ≤ 0.05, B & C-*p* ≤ 0.0001; LDL-C–A – ns, B & C-*p* ≤ 0.0001; VLDL-C: A-*p* ≤ 0.05, B & C-*p* ≤ 0.0001; TG: A-*p* ≤ 0.01, B & C-*p* ≤ 0.0001. Between groups (ANOVA and Tukey's post hoc test was done): TC: B vs A & C vs B - *p* ≤ 0.01, C vs A-*p* ≤ 0.001; HDL-C: B vs A – ns, C vs A & C vs B-*p* ≤ 0.001; LDL-C: B vs A-*p* ≤ 0.05, C vs A-*p* ≤ 0.001, C vs B-*p* ≤ 0.01; VLDL-C: B vs A-*p* ≤ 0.05, B vs C & C vs A-*p* ≤ 0.001, C vs B-*p* ≤ 0.01; TG: B vs A-*p* ≤ 0.01, C vs A & C vs B-*p* ≤ 0.001.

## Data Availability

The data will be handled on a case-by-case basis as per institutional policy.

## Abbreviations

ADR: Adverse drug reaction
ATP III: Adult treatment panel III
CRP: C-reactive protein
CV: Cardiovascular
CVD: Cardiovascular disease (CVD)
DM: Diabetes mellitus
GSH: Glutathione
HDL-C: High-density lipoprotein cholesterol
hsCRP: High sensitivity C-reactive protein
LDL-C: Low-density lipoprotein cholesterol
MDA: Malondialdehyde
MetS: Metabolic syndrome
NO: Nitric oxide
RI: Reflection index
ROS: Reactive oxygen species
TC: Total cholesterol (TC)
TG: Triglycerides
*ω*-3FA: Omega-3 fatty acids.
